# Inhibitory Effects of Mongolian Medicine Yihe-Tang on Continuous Darkness Induced Liver Steatosis in Zebrafish

**DOI:** 10.1155/2022/5794655

**Published:** 2022-05-20

**Authors:** Rigaiqiqige Sa, Chi Feng, Hongxia Bai, Xiaoyu Yin, Lei Song, Xiaodong Hu, Rui Xu, Xinshan Li, Wu Dong, Jingfeng Yang

**Affiliations:** Inner Mongolia Key Laboratory of Toxicant Monitoring and Toxicology, College of Animal Science and Technology, Inner Mongolia Minzu University, Tongliao, Inner Mongolia 028000, China

## Abstract

The constant dark induction (DD) causes lipid degeneration and nonalcoholic fatty liver disease (NAFLD) in zebrafish, which might be closely related to the imbalance of gut microbiota and require in-depth study. In this study, a total of 144 zebrafish were divided into four groups, including the control group, Yihe-Tang group, constant dark group, and constant dark + Yihe-Tang group, and were treated with constant darkness (except control and Yihe-Tang groups) for 21 days. The bodyweights of zebrafish were recorded after 8 d, 15 d, and 22 d. The sequencing analysis of gut microbiota, detection of liver histopathological changes, and comparison of lipid metabolism-related gene expression levels were performed on the 22^nd^ day of the experiment. The results showed that the Yihe-Tang could inhibit the constant dark-induced increase in zebrafish weight and liver steatosis. As compared to the control group, the dark treatment could alter the composition of gut microbiota in zebrafish, increase the relative abundance of harmful bacteria, and decrease the *Cetobacterium* and *Bacteroides* to *Firmicutes* ratio in the intestines. The abundance of Proteobacteria in the constant dark + Yihe-Tang group was close to that in the control group and that of Fusobacteria and *Cetobacterium* increased, especially the *Cetobacterium*, which increased significantly. The constant dark treatment caused an abnormal expression of liver lipid-related genes, inhibited lipid metabolism, and promoted fat accumulation. However, the Yihe-Tang could restore these changes to the level of the control group. This study indicated that Yihe-Tang could restore the constant dark-induced liver lipid degeneration. We hypothesized that *Cetobacterium* could significantly inhibit steatosis.

## 1. Introduction

In recent years, the incidence of nonalcoholic fatty liver disease (NAFLD) has been increased, causing severe health problems. NAFLD includes simple steatosis, nonalcoholic steatohepatitis (NASH), liver fibrosis, and cirrhosis [1]. It is a liver disease caused by the disorders of glucolipid metabolism and characterized by the deposition of lipids in hepatocytes. Its causative factors generally include a high-fat diet, drugs, and other factors in addition to exogenous intake of alcohol. NAFLD has now become a significant cause of chronic liver disease worldwide with a prevalence as high as 24% [2]. The effective prevention and treatment of NAFLD and NASH are needed to avoid their progression to end-stage liver diseases. Many factors, such as genetics, obesity, poor lifestyle habits, and imbalance of gut microbiota, have been reported to be highly related to the prevalence of NAFLD [3]. There is a strong correlation between gut microbiota and NAFLD [4]. In 1998, Marshall formally proposed the “gut-liver axis” concept [5]. The core function of this axis is the two-way communication between the intestine and liver, influencing each other. This means that liver can affect the intestines, especially causing changes in the composition of gut microbiota, which in turn affects the liver. Therefore, using the “gut-liver axis” as a target, the mechanism of action of the environment or drugs on NAFLD is investigated using the compositional analysis of gut microbiota. At the same time, this treatment method could also result in the significant upregulation of lipid synthesis-related genes, such as *ppar-p* and *fasn*, in zebrafish liver and also resulted in the significant downregulation of lipolysis-related genes, such as *cpt1* and *acadm*. These results indicated that the changes in gut microbiota might lead to the deposition of fats in the zebrafish liver [6].

Yihe-Tang was produced according to the “Drug Standards of the Chinese Ministry of Health (Mongolian Drugs)” and approved by the Affiliated Hospital of Inner Mongolia Minzu University (approval number: Z15021057), which is responsible for the standardization of drugs to ensure their contents. Yihe-Tang is composed of *Carthamus tinctorius* L., *Terminalia chebula* Retz., *Melia toosendan*, *Inula helenium* L., *Gardenia jasminoides J.* Ellis., *Corydalis bungeana* Turcz., *Dolomiaea souliei* (Franch.) C. Shih., *Neopicrorhiza scrophulariiflora* (Pennell) D. Y. Hong., *Gentiana macrophylla* Pall., *Ophiopogon japonicus* (L. f.) Ker Gawl., *Punica granatum* L., *Pyrus ussuriensis* Maxim., *Cyrtomium fortunei* J. Sm., *Gentiana dahurica* Fisch., *Chrysanthemum indicum* L., *Asarum heterotropoides* F. Schmidt., *Coriandrum sativum* L., *Momordica cochinchinensis* (Lour.) Spreng., *Sus scrofa domestica Brisson*, *Tussilago farfara* L., *Scabiosa comosa* Fisch., *Dianthus superbus* L., *Dracocephalum moldavica* L., *Trogopterus xanthipes*, and *Amomum kravanh* Pierre ex Gagnep. In rat models, Yihe-Tang could inhibit the alcohol-induced fatty liver by reducing the increase in glucose-deficient transferrin [7]. A study by Haosoqiqige et al. also suggested that Yihe-Tang had a therapeutic effect on the AFLD [8]. Yihe-Tang has a protective effect on the liver and has been widely used in Mongolian areas for treating liver inflammation and other liver-related diseases. However, the studies on Yihe-Tang are limited and the mechanism of action of its mixed preparation is still unclear. Several studies have reported the mechanism of action of a single decoction component in Yihe-Tang. For example, gardenoside components inhibit the NAFLD-induced oxidative stress and inflammation by upregulating the Nrf2 and modulating the protein expression and AMPK/PI3K/mTOR signaling pathway [9] or improve NAFLD by upregulating PPAR-*α* [10]. *Terminalia chebula* Retz (chebulinic acid) upregulates the Heme oxygenase-1(HO-1) and NAD(P)H quinone oxidoreductase-1(NQO1) expression levels in L-02 cells in order to resist the tert-butyl hydrogen peroxide (t-BHP-) induced oxidative stress [11]. The safflower yellow (SY) component inhibits inflammatory response, promotes collagen degradation, and regulates gut microbiota [12]. Based on the previous studies' results, Yihe-Tang might have an antioxidative stress effect or play a protective role by regulating the gut microbiota. In this study, an NAFLD animal model of zebrafish was established and the regulatory effects of Yihe-Tang on the gut microbiota of zebrafish were studied.

Zebrafish (*Danio rerio*) is a widely used experimental animal model. It has the advantages of being small and easy to raise, fast growth and development, high breeding function, and drug sensitivity. In particular, the circadian rhythm and characteristic sleep state of zebrafish are similar to those of mammals and its genetic background is highly similar to humans [13]. In this study, zebrafish were used as experimental animal models. Liver steatosis was induced through the dark environment treatment and their body weight, histological changes in liver, changes in the composition of gut microbiota, and expression of lipid metabolism-related genes were determined. The effects of Yihe-Tang on the recovery of the changes in zebrafish's gut microbiota and liver metabolism caused by the dark environment were further investigated.

## 2. Material and Methods

### 2.1. Medicines and Materials

Yihe-Tang was purchased from the Affiliated Hospital of Inner Mongolia Minzu University (approval number: Z15021057). The total RNA extraction kit, reverse transcription kit, and other drugs were purchased from Sigma-Aldrich (St. Louis, MO).

### 2.2. Feeding and Light/Dark Cycle of Zebrafish

Zebrafish (AB line) were used as experimental animals. The breeding zebrafish were maintained at 28 ± 1°C in the circulating zebrafish aquacultures (Aisho, Beijing, China) with a light/dark cycle of 14/10 h. The 4-month-old zebrafish (*n* = 144) were taken from the 12 aquariums, respectively (12 zebrafish were placed in each tank according to the male to female ratio of 1 : 1). These fish tanks were randomly assigned to each experimental group (*n* = 3 per group) and labeled as control, constant dark, Yihe-Tang, and constant dark + Yihe-Tang groups. Yihe-Tang was administered by feeding. First, the diet was supplemented with 3.5 mg Yihe-Tang/g of basal diet (the drug content was about 0.35% of the feed weight). The daily diet intake of zebrafish was calculated to be 4% of their total body weight and their average body weight was 0.28 ± 0.003 g. The daily feeding diet was 0.0112 ± 0.00012 g and the average daily dose of Yihe-Tang per fish was 39.2 ± 0.42 *μ*g. The feed was given daily at 8:00 am and 4 pm for 26 days. The first five days were considered preparatory experiment time and the next 21 days as formal experiment periods. For creating a dark environment, all the fish tanks were placed in a dark box. The feed was given gradually during the pretest period in order to ensure that the zebrafish consumed all the feed. Also, the feed residuals were checked during changing the water in the main experiment. The daily feeding rate was calculated as follows: daily feeding rate (%/d) = 100 × total feed consumed/days × (initial body weight + final body weight)/2 [14].

### 2.3. Hematoxylin-Eosin Staining (H&E) and Oil Red O (ORO) Staining of the Liver Tissues

The zebrafish in each group were treated for 8 d, 15 d, 22 d, and their livers were collected and fixed with 4% paraformaldehyde (PFA) for 16 h. Then, the livers were rinsed with phosphate-buffered saline (PBS) and dehydrated with 60, 70, 80, 90, 95, and 100% ethanol. The ethanol was then replaced by 100% for paraffin embedding. The prepared tissue sections were then stained with H&E for microscopic observation and analysis. Similarly, for the ORO staining, the liver tissues were fixed with 4% PFA for 16 h and the iced tissue sections were prepared by optimal cutting temperature (OCT) embedding. After preparation, the tissues were rinsed with 60% propylene glycol, stained with ORO stain, counterstained with sappanwood, sealed with glycerin gelatin, and observed under a microscope.

### 2.4. Sample Collection and Sequencing of Gut Microbiota

The livers and intestines were obtained using sterile surgical instruments on a clean bench. The livers were immediately put into a cryotube for RNA extraction. A sterile cotton swab was dipped into the intestinal contents of the intestines and immediately put into a cryotube, which was then stored in liquid nitrogen in a refrigerator at −80°C and used for *16S rRNA* gene sequencing (*n* = 6). The *16S rRNA* gene sequencing was performed by Nuovo Zhiyuan (Beijing, China). The genomic DNA (gDNA) from each sample was extracted using the sodium dodecyl sulfate (SDS) method and the polymerase chain reaction (PCR) amplification was performed using specific primers (barcode label), template DNA (1 ng/*μ*l), and Phusion® high-fidelity PCR Master Mix (New England Biolabs, USA). For the identification of gut microbiota, the following gene regions were amplified: V3-V4 hypervariable region of *16S rRNA* gene for the identification of gut bacterial diversity, V4 hypervariable region of *18S rRNA* gene for the identification of gut eukaryotic microbial diversity, and *ITS1* region for the identification of gut fungal diversity, 16S V3-V4/16S V4-V5/16SV5-V7, Archaea 16S V4-V5/Archaea 16S V8, 18S V9, and *ITS2* gene regions. The amplified PCR products were detected on 2% agarose gel and the target bands were recovered using a gel recovery kit (QIAGEN, Germany). The sequencing library was constructed with a TruSeq® DNA PCR-Free Sample Preparation Kit (Illumina, USA). Qubit and q-PCR were used for the quantification of library and the qualified libraries were sequenced using a NovaSeq 6000 sequencing platform (Illumina, USA).

### 2.5. RNA Extraction and qRT-PCR Analysis

According to research [6], TRIzol reagent (Ambion, USA) was used to extract the total RNA from zebrafish liver tissues. A total of 250 ng of the extracted RNA samples with OD 230/260 and OD 260/280 of greater than 1.8 were reverse transcribed to obtain cDNA. The TB Green Premix Ex Tax II kit was used for qRT-PCR analysis. The primer sequences used in the study are listed in Table 1 and 18S was used as an internal reference gene, and the changes in the relative expression of genes were calculated using the 2^ΔΔCt^ method.

### 2.6. Statistical Analyses

One-way analysis of variance (ANOVA) and *t*-test were used for the data analysis and the significance threshold level was set to *P* < 0.05. All the data were expressed as mean ± standard error of the mean (SEM).

## 3. Results

### 3.1. Effects of Yihe-Tang on the Weight Gain of Zebrafish Induced by the Dark

**Figure 1:**
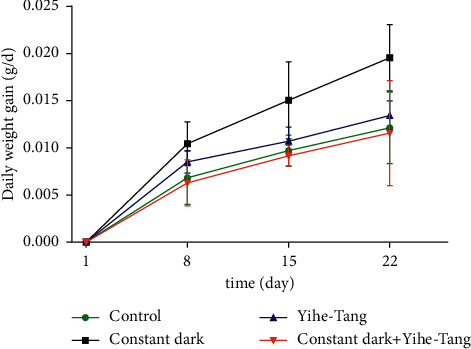
Effects of Yihe-Tang on the constant dark-induced weight gain of zebrafish. Experimental setup included four groups; control, Yihe-Tang, constant dark, and constant dark + Yihe-Tang group. The bodyweights of zebrafish were measured on 8 d, 15 d, and 22 d of the treatment (*n* = 25 d).

In order to analyze the effects of Yihe-Tang on the weight gain of zebrafish induced by the constant darkness, their body weights were measured on 8 d, 15 d, and 22 d of the treatments. The results showed that the average daily weight showed an increasing trend in the constant dark group which tended to increase in each period. As compared to the control group, the average daily weights of zebrafish in the constant dark group increased by 53%, 54%, and 62%, respectively, while those in the constant dark + Yihe-Tang group returned to the control level (Figure 1).

### 3.2. Effects of Yihe-Tang on the Pathological Changes of Liver Tissue Induced by the Constant Darkness in Zebrafish

**Figure 2:**
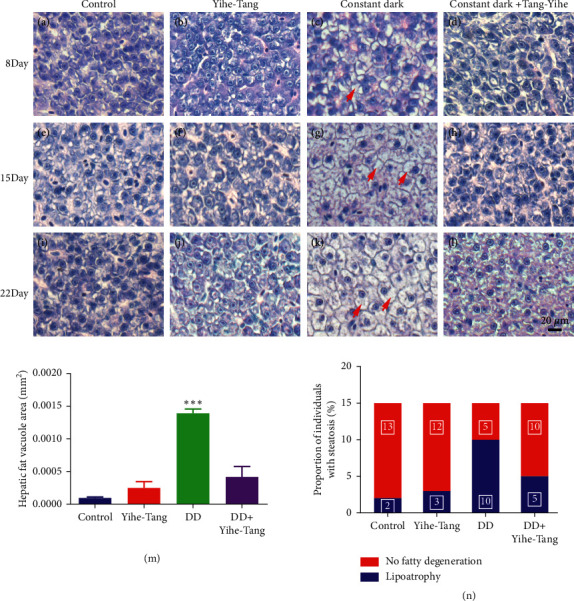
Effects of Yihe-Tang on the degeneration of fats in the zebrafish liver tissues induced by the constant darkness. ImageJ software was used to quantify the liver vacuole area. Control group: (a), (e), and (i); Yihe-Tang group: (b), (f), and (j); constant dark group: (c), (g), and (k); constant dark + Yihe-Tang group: (d), (h), and (l); (m): quantification of void area, (n): fatty degeneration scale, *n* = 15, scale bar = 20 *µ*m.

After the 8 d, 14 d, and 22 d of constant dark induction, the zebrafish livers were collected for H&E staining. The results showed that the zebrafish liver had no significant changes in each period. The liver cells were regularly arranged with dense chromosomes and visible nuclei in the control and Yihe-Tang treatment groups. However, in the constant dark group, the zebrafish liver showed a small amount of steatosis after 8 d and severe steatosis after 14 and 22 d of constant dark treatment (Figures 2(g) and 2(k)). As compared to the control group, the vacuolation of fish liver tissue increased by 13.93 times (*P* < 0.05) in the constant dark group (Figure 2(m)). After the Yihe-Tang treatment (constant dark + Yihe-Tang group), the zebrafish liver tissues did not show a significant increase in the degeneration of fats and vacuoles after the 8 d and 15 d of constant dark treatment. However, the degeneration of fats and vacuoles in the liver at 22 d was significantly lower in the constant dark + Yihe-Tang group than that in the constant dark group (*P* < 0.05); the fats were 30% of those in the dark treatment group (Figure 2(d)). The statistical results showed that the constant dark + Yihe-Tang group significantly reduced the constant dark-induced steatosis (Figures 2(m) and 2(n)).

### 3.3. Improving Effects of Yihe-Tang on the Hepatic Fat Accumulation Induced by the Constant Darkness in Zebrafish

**Figure 3:**
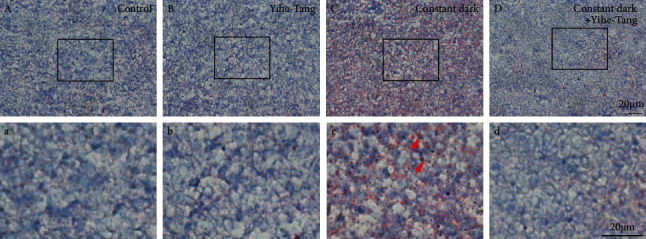
Improving effect of Yihe-Tang on the constant dark-induced liver fat accumulation in zebrafish. The zebrafish were divided into control, constant dark, Yihe-Tang, and constant dark + Yihe-Tang groups, after 21 d of treatment (*n* = 3). Their liver tissues were collected and stained with Oil Red O to detect the histological changes in the liver of zebrafish. *N* = 3, scale bar = 20 *µ*m.

The ORO staining results showed that there was no lipid accumulation in the control group. As compared to the control group, a large number of red lipid droplets were accumulated in the constant dark-induced group. However, the accumulation of lipid droplets in the constant dark + Yihe-Tang group improved significantly as compared to the constant dark group, as shown in Figure 3. The results showed that the Yihe-Tang could improve the fat accumulation induced by the constant dark treatment.

### 3.4. Effects of Yihe-Tang on the Dark-Induced Changes in Gut Microbiota

**Figure 4:**
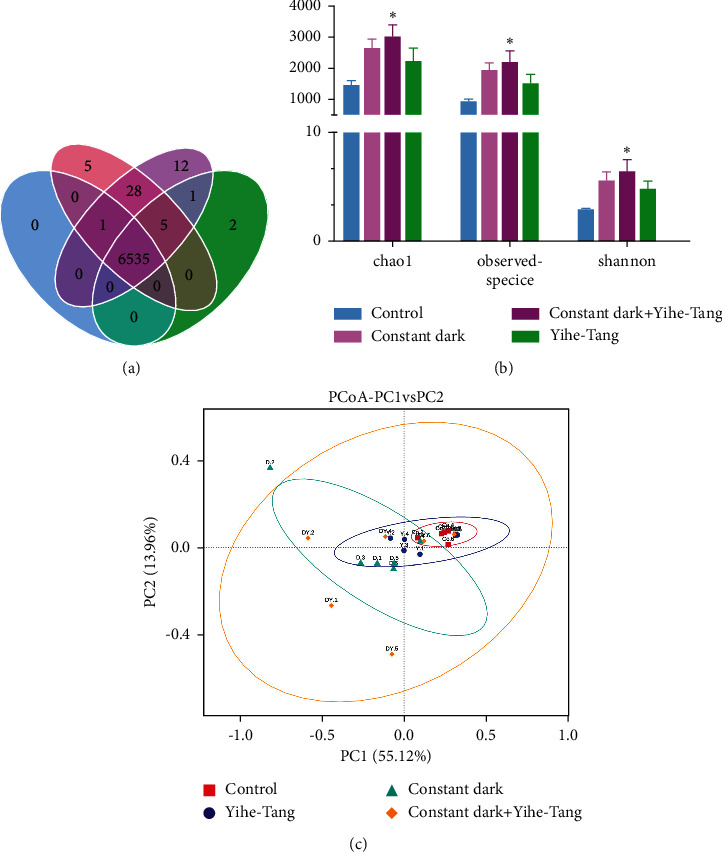
Effects of Yihe-Tang on the changes of zebrafish gut microbiota caused by the constant dark treatment. (a) Venn diagram summarizes the number of standards and different OTUs. (b) Microbial diversity index. (c) PCoA analysis. The horizontal and vertical axes in (c) represent the different principal components, and the percentage represents the contribution value of the main element to the sample difference. The data are expressed as mean, standard error (SE) of the mean (*n* = 6). “^*∗*^” indicates a significant difference (*P* < 0.05).

A comparative analysis of the gut microbiota of the four different treatment groups was performed based on *16S rRNA* gene sequencing technology. A total of 80563 raw sequence reads were obtained from the 24 gut samples, among which 76355 high-quality sequence reads were obtained after the screening and filtering for subsequent analysis. A threshold of 0.97 was selected for the clustering analysis and comparison, resulting in a total of 9,321 operational taxonomic units (OTUs). About 99.2% of these microbes were clustered into common phyla against the four treatment groups (Figure 4(a)). The alpha diversity indices (Shannon and Simpson indices) of the zebrafish gut microbiota increased significantly (*P* < 0.05) in the constant dark + Yihe-Tang group as compared to the control group, while the species and chao1 indices were also significantly higher (*P* < 0.05) (Figure 4(b)). Principal coordinate analysis (PCoA) plots showed that the clustering of microbial communities in each group was different (Figure 4(c)).

**Figure 5:**
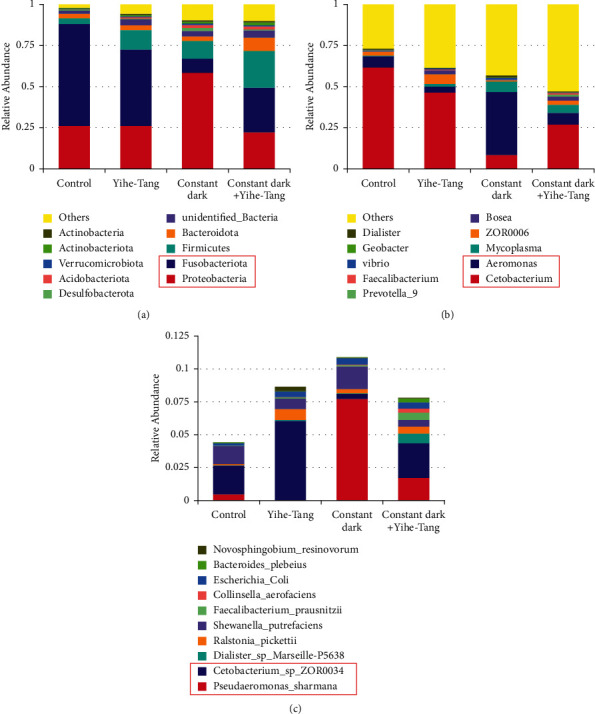
Effect of Yihe-Tang on the relative abundance of gut microbiota in zebrafish. (a) Relative abundance at the phylum level, (b) relative abundance at the genus level, and (c) relative abundance at the species level. The data of the top ten microbial communities with the highest relative abundances were selected from the above four groups.

Based on the results of OTU species annotation, the top ten species with the highest abundance were selected and classified in the phyla, orders, families, and genera and were analyzed comparatively using the cumulative plots of the species relative abundance. The analysis at the phylum level revealed that there was no significant difference in the gut microbiota of Yihe-Tang group as compared to the control group; the relative abundance of phylum Proteobacteria increased significantly in the dark group, while that of *Clostridium* (Fusobacteria) decreased significantly. The relative abundance of Proteobacteria in the constant dark + Yihe-Tang group was similar to that in the control group, while the relative abundance of *Fusobacteria* was significantly reduced in the constant dark + Yihe-Tang group but tended to increase as compared to the constant dark group (Figure 5(a)). At the genus level, the highest relative abundance of *Cetobacterium* was found in the Yihe-Tang group, which was a dominant species in this treatment group, while its abundance was significantly reduced in the constant dark group. Interestingly, the relative abundance of *Cetobacterium* significantly increased in the constant dark + Yihe-Tang groups (Figure 5(b)).

**Figure 6:**
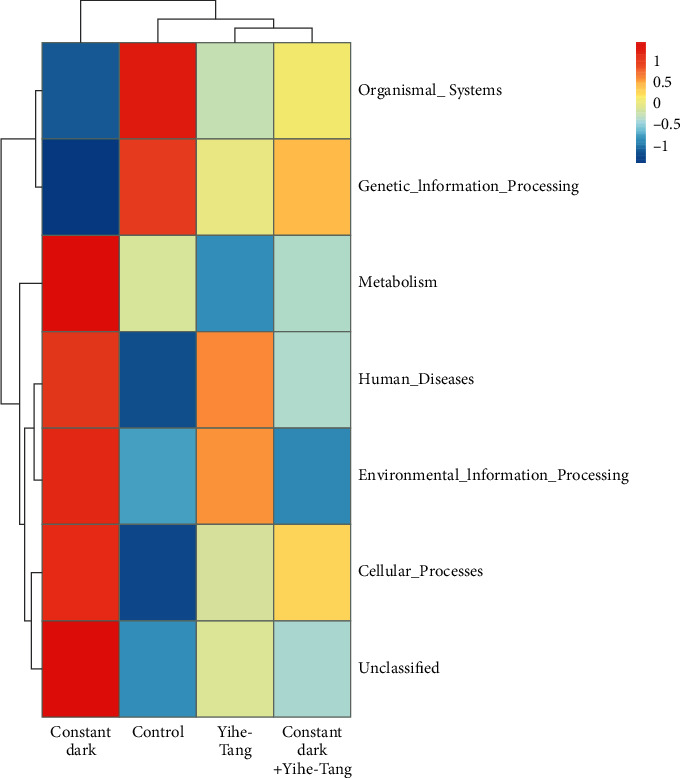
Clustering heat map for predicting the function of gut microbiota. The red color indicates that the abundance of gut microbiota was large or the corresponding annotation function was enhanced and vice versa for the blue color. Four groups were analyzed and compared and the microbial colonies with the highest abundances were selected for drawing.

The prediction of microbial functions based on the dominant gut microbiota in each treatment group showed that the functions of microbiota, including metabolic function, cellular transformation, human disease, and environmental information feedback, were higher in the constant dark group as compared to the control group. Two functions, including the organic system and genetic information feedback, were lower in the constant dark group than those in the control group. The Yihe-Tang treatment could restore all these functional microbiotas (Figure 6).

### 3.5. Effect of Yihe-Tang on the Expression of Lipid Metabolism-Related Genes

**Figure 7:**
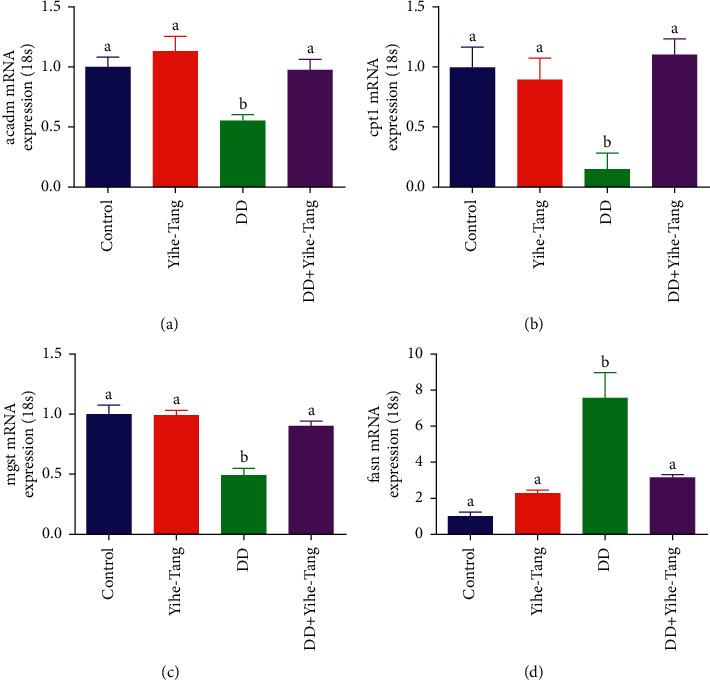
Yihe-Tang restored the constant dark-induced changes in the expression levels of lipid metabolism-related genes. Zebrafish were treated with dark in the control, Yihe-Tang, constant dark, and constant dark + Yihe-Tang groups for 22 days and the liver tissues were collected for RT-PCR analysis. (a): *acadm*, (b): *cpt1*, (c): *mgst*, and (d): *fasn*. Data are expressed as mean ± standard error (SEM) (*n* = 9), and different letters indicate significant differences (*P* < 0.05).

When the zebrafish were treated with and without Yihe-Tang in a constant dark for 22 days, the expression levels of the genes associated with fat metabolism were analyzed using RT-PCR. The results showed that constant dark induction significantly reduced the mRNA expression levels of *cpt1, acadm,* and *mgst*, which decreased by 15%, 55%, and 48% (Figure 7(a)–7(c)), respectively as compared to the control group (*P* < 0.05). On the contrary, the constant dark induction increased the mRNA expression level of *fasn* by 7.6 times as compared to the control group (*P* < 0.05) (Figure 7(d)). However, the gene expression levels in the constant dark + Yihe-Tang group were recovered to the control level (*P* < 0.05).

## 4. Discussion

The constant dark environment for a long time causes liver steatosis in zebrafish, which can be relieved after treatment with Yihe-Tang. In this study, the analysis of gut microbiota in zebrafish showed that the constant dark induction increased the abundance of harmful gut microbiota and significantly decreased the relative abundance of genus *Cetobacterium*. In contrast, the treatment with Yihe-Tang could significantly restore the abundance of genus *Cetobacterium*. We hypothesized that *Cetobacterium* could significantly inhibit steatosis. At the genetic level, the constant dark treatment caused significant changes in the expression levels of lipid metabolism-related genes. However, Yihe-Tang also significantly restored this change. The experimental results indicated that the changes in steatosis and gene regulation caused by the constant dark treatment might be related to the changes in the gut microbiota of zebrafish.

Chen et al. used Chinese herbal medicine (919 syrup) in the NAFLD rat models and showed a reduction in the weight of rats and improvement in the liver histopathological changes caused by NAFLD [15], which was consistent with the present study. Hung et al. found that Pingtang No. 5 Capsule (PT5, a traditional Chinese medicine compound of Alisma Decoction) could inhibit the accumulation of liver lipids and liver cell damage and reduce the bodyweight in NAFLD mice [16].

In order to study the inhibitory effect of Yihe-Tang on fat accumulation, Uzhitunashun et al. showed that Yihe-Tang inhibited the alcohol-induced abnormal apoptosis of rat liver cells, thereby improving the liver function [17]. In fatty acid synthesis, *fasn* is a downstream gene in the srebp1 pathway and a key fat synthesis enzyme, which is closely related to the production and accumulation of fat. The *cpt1* gene plays a key role in lipolysis *β*-oxidation. The *acadm* gene is a key lipolytic gene [18]. The constant dark induction caused a significant decrease in the mRNA expression levels of *cpt1* and *acadm* genes, indicating that the dark had a low effect on fat degradation. The Yihe-Tang treatment could significantly upregulate the expression levels of *cpt1* and *acadm*, indicating that the treatment with Yihe-Tang could promote the fat degradation function of the liver in zebrafish. The *fasn* is an important gene, regulating the fatty acid synthase, which is involved in the entire process of fatty acid synthesis. The constant dark treatment significantly increased the mRNA expression level of *fasn*, which was restored by Yihe-Tang treatment. In addition, the *mgst* is a decomposing enzyme related to oxidative stress metabolites [19]. It is highly expressed in various organs. Its antioxidant effects can protect liver cells [20–22]. The present study showed that the constant dark induction could reduce the expression level of *mgst* gene. At the same time, the Yihe-Tang could significantly increase the expression level of *mgst* gene, indicating that Yihe-Tang could enhance the liver's ability to break down lipids.

Feng et al. showed that the constant dark induction could significantly affect the gut microbiota and liver metabolism in zebrafish and indicated that the changes in gut microbiota could affect the liver metabolism in zebrafish [6]. Since the liver is connected to the gut microbiota through the “gut-liver axis,” the pathogenesis of NAFLD is closely related to the gut microbiota, which has been a hot research topic in recent years. In this study, *16S rRNA* gene sequencing technology was used to detect the effects of Yihe-Tang on the gut microbiota of zebrafish. The effects of Yihe-Tang on the gut microbiota of zebrafish under dark treatment were observed. As compared to the control group, the constant dark induction could reduce the ratio of Firmicutes to Bacteroides (F/B) in the gut microbiota of zebrafish, while the Yihe-Tang treatment could significantly increase the richness of gut microbial community. The F/B ratio also increased. A study showed that the constant dark environment could cause a significant decrease in the proportion of Bacteroides and Firmicutes in the zebrafish intestines, thereby leading to metabolic disorders, fat accumulation, and weight gain in zebrafish [5]. These gut bacteria are related to obesity and diabetes, as well as energy absorption [23]. The F/B ratio can be used as an indicator of obesity [24]. In this study, the relative abundances of Fusobacteria were 7.38 times and 2.38 times lower in the constant dark and Yihe-Tang groups, respectively, as compared to the control group.

The short-chain fatty acids (SCFAs) are metabolites of *Cetobacterium* and regulate lipid metabolism as a substrate for lipid synthesis [25]. The correlation between SCFAs and NAFLD has been focused on in recent years. Studies have shown that the regulation of SCFAs by *Cetobacterium* plays an important role in maintaining the health of zebrafish intestines [26]. In this study, as compared to the control group, the constant dark induction could reduce the abundance of *Cetobacterium* in the zebrafish intestines and cause lipid degeneration in the liver. However, the intervention of Yihe-Tang increased the abundance of *Cetobacterium* and inhibited steatosis. The result indicated that Yihe-Tang might exert an anti-inflammatory effect by regulating the production of SCFAs by *Cetobacterium*. *Cetobacterium*, accounting for 70% of the fish gut microbiota, is a rod-shaped Gram-negative anaerobic bacterial genus, which ferments SCFAs, including acetate, propionate, and butyrate [14, 27], among which acetate is the main fermentation product [28]. Canfora et al. reported that both the acetate and propionate increased the plasma acetate concentrations (fasting condition), promoted fat oxidation, and decreased the circulating free glycerol concentrations after the rectal administration of acetate or propionate during fasting and postprandial conditions (oral glucose load) [29]. In addition, acetate could also affect appetite by secreting the gut hormones, such as glucagon-like peptide 1 and peptide YY, in order to enhance host energy and substrate metabolism, increase fat oxidation and energy expenditure, and reduce systemic fat synthesis [30]. Therefore, restoring the abundance of *Cetobacterium* can effectively supplement acetate, thereby effectively inhibiting fat accumulation and steatosis.

Circadian clocks regulate metabolism and energy homeostasis in the liver and other peripheral tissues [31]. Human and rodent studies have clearly shown that hepatic fat accumulation and steatosis are closely associated with insulin resistance [32]. In the insulin-resistant states, insulin loses its ability to inhibit the production of glucose, while still promoting hepatic lipid synthesis even in a hyperactive state [33]. Perry et al. reported that acetate could activate the parasympathetic nervous system, which in turn promoted the secretion of glucose-stimulated insulin [34]. Consistent with mammalian findings, the addition of acetate to the diet promoted insulin secretion and glucose utilization in zebrafish, thereby suggesting a conservative role of acetate in regulating glucose homeostasis [14]. In adult zebrafish, the abundance of *Cetobacterium* and intestinal acetic acid levels were highly correlated, suggesting that the positive effects of *Cetobacterium* on glucose homeostasis in zebrafish were mediated by the production of acetic acid [31]. This suggested that the acetate-brain-insulin secretion axis is conserved between fish and mammals, revealing a specific intestinal “*Cetobacterium*-brain” pathway, which regulates glucose homeostasis in zebrafish [14].

## 5. Conclusions

Yihe-Tang could inhibit the increase in zebrafish weight caused by the constant dark induction and inhibit lipid degeneration in the zebrafish liver cells. This inhibition was closely related to the recovery of zebrafish gut microbiota. We hypothesized that *Cetobacterium* could significantly inhibit steatosis. For example, Yihe-Tang reduced the abundances of the phylum Proteobacteria, increased those of the phylum Fusobacterium and genus *Cetobacterium*, and increased the F/B ratio. At the same time, the constant dark induction caused an abnormal expression of liver lipid metabolism-related genes, which was reversed by Yihe-Tang. This study showed that the recovery effects of Yihe-Tang on the dark-induced liver metabolism disorder in zebrafish were closely related to the recovery of *Cetobacterium*.

## Figures and Tables

**Table 1:** The primers for RT-qPCR.

Gene	Primer sequences (5′-3′)	Forward/Reverse
18s	TCGCTAGTTGGCATCGTTTATG	Forward
ID: 100037361	TCGCTAGTTGGCATCGTTTATG	Reverse
fasn	GCACCGGTACTAAGGTTGGA	Forward
ID: 559001	ACACAACCGACCATCTGTCA	Reverse
acadm	AGGTTTTGAGGGCAGGTGTT	Forward
ID:406283	TCTGCTGCTCGGTTAGTTCA	Reverse
cpt1	ATGAGGAGCACCAAAGAATG	Forward
ID: 122874692	TGGGAAAAGCGTAAAGAAAG	Reverse
mgst	GATATGTGGCGCTAACCGGA	Forward
ID: 449784	ATGCTGAATCCCACCCACAG	Reverse

## Data Availability

The study data are available from the corresponding author upon request.
